# NIST Calibration of a Neutron Spectrometer ROSPEC

**DOI:** 10.6028/jres.111.032

**Published:** 2006-12-01

**Authors:** Craig Heimbach

**Affiliations:** National Institute of Standards and Technology, Gaithersburg, MD 20899

**Keywords:** calibration, neutron, NIST, ROSPEC, spectroscopy

## Abstract

A neutron spectrometer was acquired for use in the measurement of National Institute of Standards and Technology neutron fields. The spectrometer included options for the measurement of low and high energy neutrons, for a total measurement range from 0.01 eV up to 17 MeV. The spectrometer was evaluated in calibration fields and was used to determine the neutron spectrum of an Americium-Beryllium neutron source. The calibration fields used included bare and moderated ^252^Cf, monoenergetic neutron fields of 2.5 MeV and 14 MeV, and a thermal-neutron beam. Using the calibration values determined in this exercise, the spectrometer gives a good approximation of the neutron spectrum, and excellent values for neutron fluence, for all NIST calibration fields. The spectrometer also measured an Americium-Beryllium neutron field in a NIST exposure facility and determined the field quite well. The spectrometer measured scattering effects in neutron spectra which previously could be determined only by calculation or integral measurements.

## 1. Introduction

A Bubble Technology Industries (BTI) Rotating Spectrometer^1^ (ROSPEC) Ref. [1], was purchased by the National Institute of Standards and Technology (NIST) for use in applications supporting the Department of Homeland Security (DHS). NIST applications include the validation of the spectra of calibration sources and an evaluation of whether a neutron spectrometer could distinguish, through the measurement of neutron spectra, potential threat sources from innocuous sources.

ROSPEC has the unique ability to measure neutron spectra from 0.01 eV to 17 MeV with the spectrum above 50 keV having resolution of 10 % to 15 %. Currently, any neutron source of interest to Homeland Security will fall within this energy range.

Various configurations of ROSPEC exist. The configuration acquired by NIST includes seven detectors. There are two ^3^He detectors, which determine the neutron spectrum from 0.01 eV to 50 keV. This part of the spectrum is given in three groups, 0.01 eV to 1 eV, 1 eV to 10 keV, and 10 keV to 50 keV. There are four gas-filled proton-recoil proportional counters which measure the spectrum from 50 keV to 4.5 MeV. There is a plastic scintillator proton-recoil detector which measures the spectrum from 4 MeV to 17 MeV. The energy spans of the various detectors overlap, providing some redundancy and consistency checks for the various detectors.

## 2. Description of Detector System

Currently, ROSPEC comes in three variants. The base system consists of four gas-filled proton recoil detectors which rotate about a common axis. Each detector is optimized to cover a particular energy range while being able to reject gamma rays because of pulse height. The total energy covered by the base system is 50 keV to 5 MeV.

The base system is supplemented by two options. One is a set of paired bare/boron-covered ^3^He detectors, which has sensitivity to neutrons down to thermal neutron energy. The other is a Simple Scintillation Spectrometer (SSS), several small plastic scintillators coupled to a photomultiplier tube, sensitive up to 17 MeV. The ^3^He detectors are integrated into the base ROSPEC, while the SSS comes as a separate package. NIST purchased both options to be able to measure all neutron fields of interest to DHS.

**Fig. 1:**
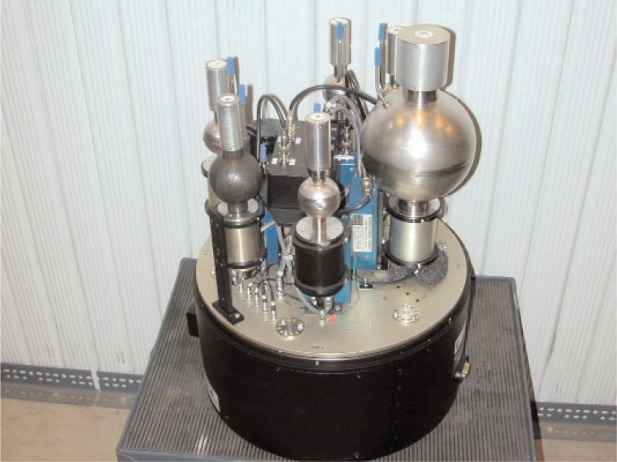
Base ROSPEC with six proportional counters.

Figure 1 shows the NIST system with its cover removed. The detectors are listed in Table 1. The largest detector, SP6, is the sphere on the right side of the picture. The boron-covered ^3^He is the black sphere in the picture. The bare ^3^He is mounted opposite the boron-covered detector to minimize interference with the opposite detector. In the picture it is behind the SP6.

**Fig. 2:**
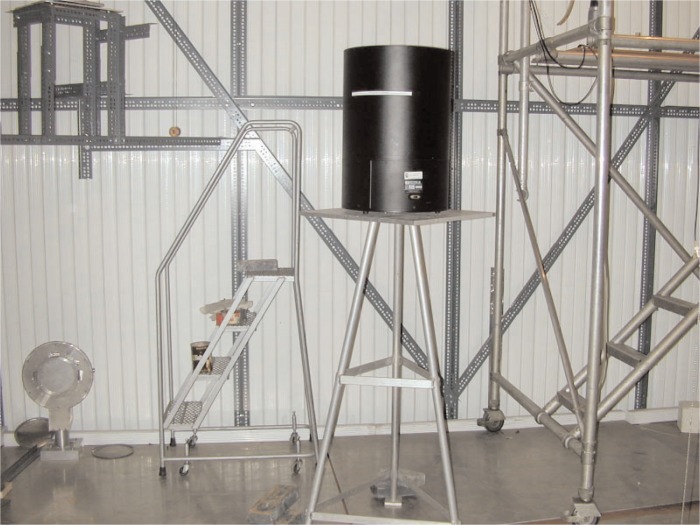
ROSPEC with cover on.

**Fig. 3:**
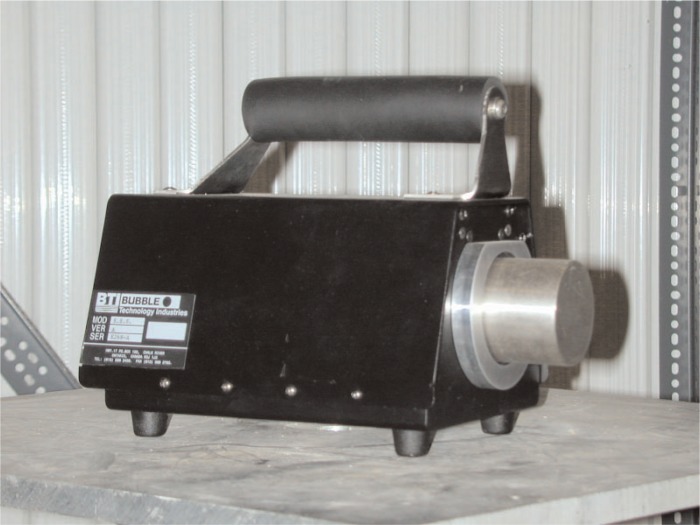
Simple Scintillator Spectrometer (SSS) detector.

Figure 2 shows ROSPEC with its normal protective cover. Figure 3 shows the SSS.

In operation, the ROSPEC detectors rotate horizontally about an axis through the center of the cylinder at approximately 4 revolutions per minute. This averages out local variations in the neutron field. All of the ROSPEC electronics are located within the cylinder. It is connected to a control laptop computer through a 30 m long cable. The size of the package is 41 cm diameter by 60.5 cm high, with a mass of about 23 kg.

The SSS consists of a detector and a control computer. The detector mass is approximately 3 kg, with an overall length of 28 cm. The control computer is an Atari portfolio which is connected to the detector with a 1.2 m long cable.

The neutron spectra must be analyzed, or unfolded, from the measured data. The ROSPEC data may be unfolded directly on the control computer. The SSS data must be downloaded through a parallel port on the Atari to the ROSPEC control computer for analysis. The provided software combines the ROSPEC and SSS data to derive a neutron spectrum. The unfolding above 50 keV is based on the SPEC4 code [2], which is a spectrum stripping code. The response at any energy is corrected for response to higher-energy neutrons by stripping off their effects. Derivatives of the data and response functions are used to avoid accumulation of errors. The spectrum below 50 keV is obtained with a three-energy-group fit to the bare and boron-covered ^3^He data, joined to the spectrum from the higher energy detectors.

## 3. Calibration Fields

NIST has several calibration fields useful for validating the performance of ROSPEC. These include bare [3] and heavy-water (D_2_O) moderated ^252^Cf sources [4,5], a thermal-neutron beam, and 2.5 MeV and 14 MeV sources. The 2.5 MeV and 14 MeV sources are of known energy, but NIST is still calibrating their output intensity. These sources span the energy range of ROSPEC.

Two of these sources, the bare and moderated ^252^Cf, are used in the NIST low-scatter facility [6]. This is a room, 11 m × 11.6 m × 9.6 m high, separate into two sections by an aluminum mezzanine. The lower section is below grade, with concrete floor and concrete walls approximately 4.6 m high. When used for calibration, either the bare or moderated source is exposed in the lower section, approximately centered in all directions. The upper section has aluminum walls and ceiling. These sources are international standard sources.

Although these two sources are well-characterized at all neutron energies, the actual spectrum is not normally used for calibration. NIST practice is to correct for the effects of room scattering in any measurement so the reported results are appropriate for pure (unscattered) fields. For calibration of spectra, the actual spectrum at 100 cm (center-to-center) was used for comparison. Monte Carlo N-Particle (MCNP) [7] calculations were performed to obtain the theoretical spectra. These calculations included air, the concrete floor and walls, and the aluminum mezzanine and upper section aluminum walls.

The NIST thermal neutron calibration facility was used to check the thermal-neutron calibration. This facility consists of a collimated beam with a line-of-sight view of a graphite moderator alongside the core of NIST research reactor. The graphite moderator is outside the heavy-water moderator of the reactor and shielded by bismuth. Thermal-neutron fluence calibration was provided with a ^239^Pu fission chamber with an overall accuracy of 5 %.

The remaining calibration sources, the 2.5 MeV and 14 MeV neutron generators are located in the NIST Californium Neutron Irradiation Facility (CNIF). The CNIF consists of a room within a room. The outer concrete walls enclose a 10 m × 15 m × 6.2 m high room. Calibrations are performed within 6.3 cm thick shell of anhydrous borax, which reduces neutron room return from the concrete. The interior dimensions of the shell are 5.2 m × 5.2 m × 5.9 m high. The neutron generators provide neutrons of known energy, but are not yet calibrated for intensity.

## 4. Calibration Measurements

This memo records the performance of ROSPEC in the various fields. Previously, NIST had calibrated a version of ROSPEC containing only the proton recoil detectors, with good results [8]. Other calibration and intercomparison results are listed in Refs. [9,10,11]. Two sets of ROSPEC calibration constants will be used to compare measurements with calibration values, one set supplied by the manufacturer and another set derived in the course of these measurements. The two sets of constants differ only in their efficiencies for detecting low-energy neutrons, i.e., only for the bare and boron-covered ^3^He detectors.

The first measurements were in the bare and heavy-water moderated ^252^Cf driven neutron fields. The best estimates of the spectra for the two fields were compared directly to measurements. These estimates were MCNP calculations of the spectrum which included scattering from air, walls, floor, and ceiling.

**Fig. 4:**
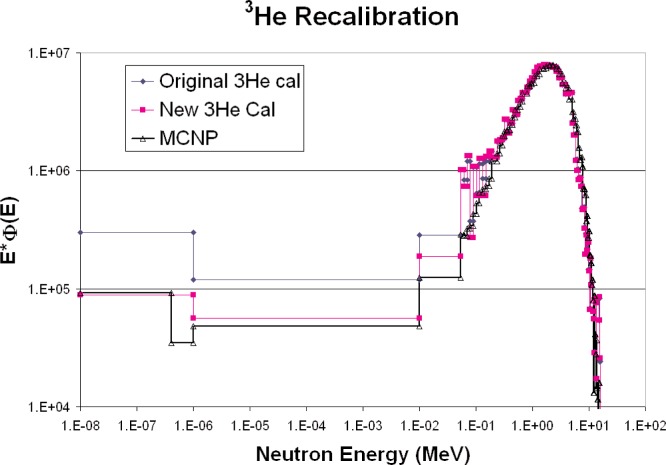
Bare ^252^Cf neutron spectrum, calculated and measurements with different calibration factors.

**Fig. 5:**
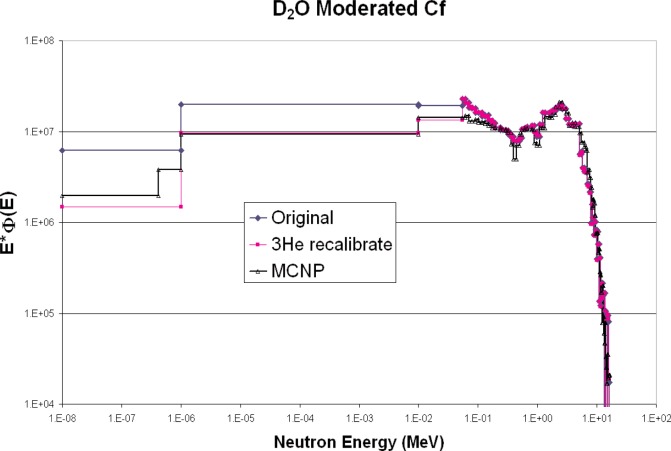
D_2_O-moderated ^252^Cf neutron spectrum, calculated and measurements with different calibration factors.

Figure 4 shows a comparison of the bare ^252^Cf versus measurement, and Fig. 5 shows a comparison of the heavy-water moderated ^252^Cf versus measurement. Above 100 keV, the agreement is quite good, especially considering that there was no normalization of spectra.

Below 100 keV, the manufacturer-supplied calibration values uniformly overestimate the value of the neutron fluence. It should be noted that the low-energy portion of the spectrum is largely due to wall scattering, and that NIST calibrations using these fields usually employ analytic and empirical corrections to remove the effects of wall scattering. Thus, the low-energy calibration.

Because of the lack of agreement between ROSPEC and calculated spectra, the NIST thermal neutron calibration facility was used to check the low-energy calibration. Only the sensitivity of the bare ^3^He detector could be measured in this facility. The sensitivity of the boron-covered detector could not be directly measured in the thermal neutron beam because boron, by design, shields the sensitive volume from thermal neutrons. The response of the boron-covered detector was determined by calculation of its sensitivity from known gas pressure and from boron attenuation. The relative response of the boron-covered versus bare ^3^He detectors as a function of energy was supplied by Bubble Technologies. Calibration results are given in Table 2.

**Fig. 6:**
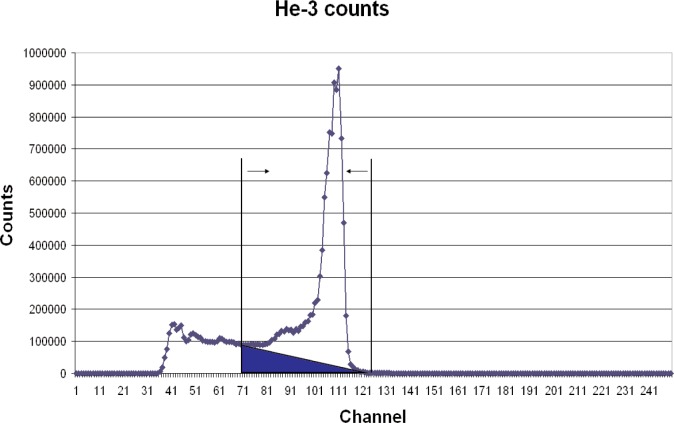
Measured ^3^He spectrum. Counts used are net counts above background between two channels.

The unfolding program used by ROSPEC uses net counts above a threshold to determine ^3^He counts (Fig. 6). It also has a scaling factor to account for neutrons which give counts below the threshold. To avoid confusion with these factors, the calibration technique used here was to insert the ^3^He counter into a known thermal neutron field and to adjust the ROSPEC calibration factor for the bare ^3^He counter until the correct unfolded thermal neutron fluence was obtained. The differences between calibration factors supplied with ROSPEC and those obtained here are substantial. The new factors determined in the NIST thermal neutron facility give a much better fit to the measured Californium spectra, as shown in Figures 4 and 5.

The National Physical Laboratory (NPL) in the United Kingdom had a similar experience with a ROSPEC they evaluated and used similar procedures to obtain their slow-neutron calibration factors [11].

**Fig. 7:**
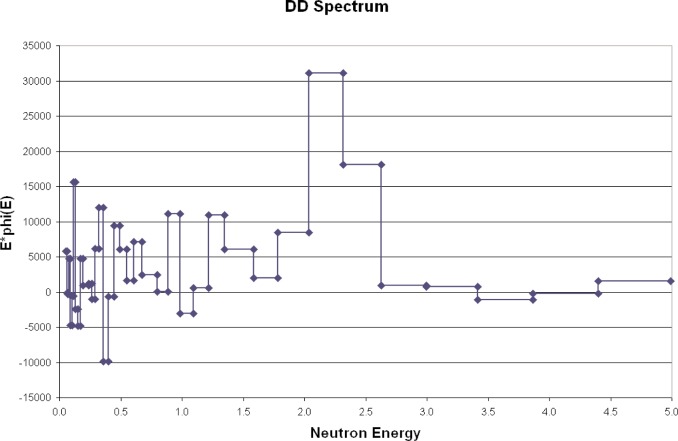
Measured DD spectrum.

**Fig. 8:**
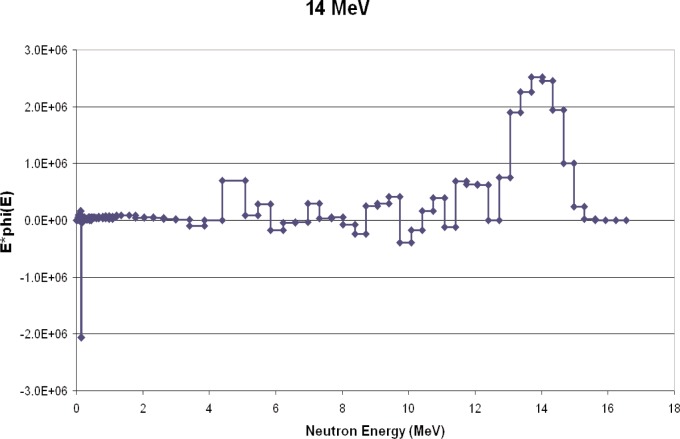
Measured DT spectrum.

ROSPEC was used to measure 2.5 MeV (deuterium-deuterium) and 14 MeV (deuterium-tritium) neutrons. As stated above, only the energies and not the intensities of the fields were known. The measured spectra are shown in Figs. 7 and 8. 2.5 MeV is in the region measured by gaseous proton-recoil detectors, and 14 MeV is in the region measured by the plastic scintillator (SSS).

In addition to verifying that ROSPEC can measure 2.5 MeV and 14 MeV neutrons at the correct energies, the measured spectra also indicate the extent to which room scattering contributes to the overall fluence. More is given on this in the next section.

## 5. AmBe Neutron Source

ROSPEC was used to measure the neutron spectrum of an AmBe neutron source. This source was chosen because such sources are commonly found in commerce and might be confused with illegitimate neutron emitters. These sources are used, among other things, in moisture density gauges.

**Fig. 9:**
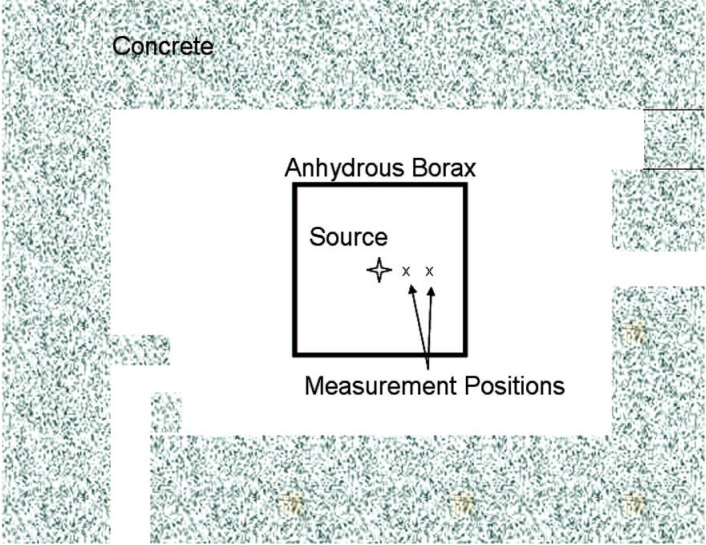
Californium Nautron Irradiation Facility (CNIF).

**Fig. 10:**
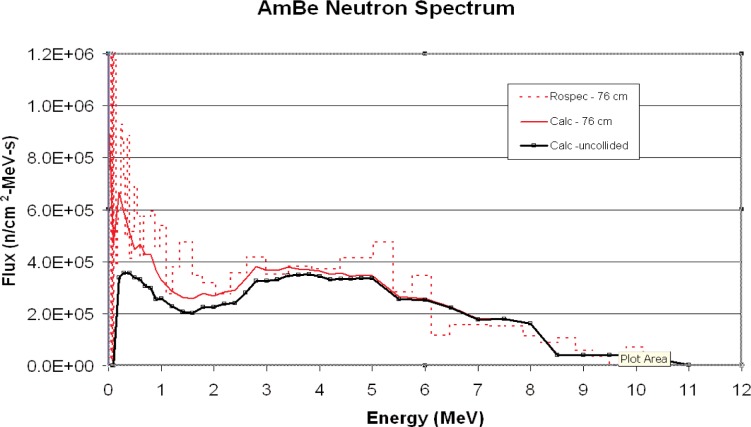
Measured and calculated AmBe spectra, 76 cm.

**Fig. 11:**
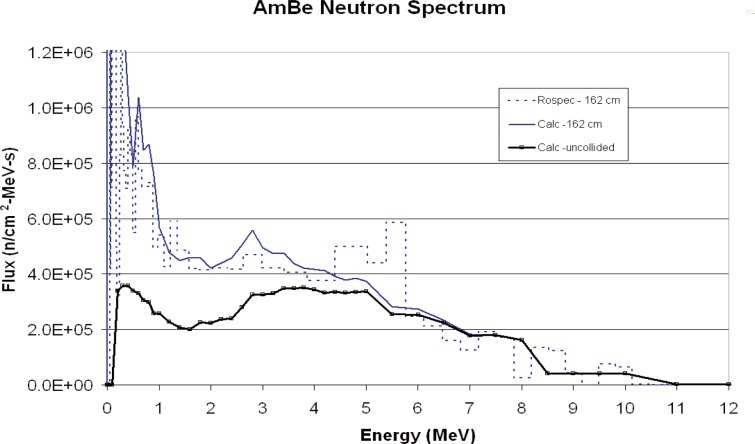
Measured and calculated AmBe spectra, 162 cm.

The neutron spectrum was measured in the NIST Californium Neutron Irradiation Facility. A drawing of the facility is given in Fig. 9. The measured and calculated spectra are shown in Figs. 10 and 11. The uncollided neutron spectrum used for calculation is taken from Ref. [12]. Actual AmBe spectra show substantial variation [13] so that the calculated spectrum used may not be a good representation of the true spectrum.

The calculated spectra show substantial scattering as the source/detector distance increases. The measured spectra show the same trend, with good overall agreement.

The ability to measure neutron spectra in this detail is a capability which ROSPEC brings to NIST.

## 6. Comparison of Integral Results

The number of measured neutrons is compared to the calibration numbers in Table 3. The calculated (no walls) column gives the calculated neutron fluence at the detector location divided by the neutron fluence from an unshielded source. For these calculations, the walls were taken to be vacuum. The units n/S_n_ indicate the number of neutrons/cm^2^ at the measurement location, multiplied by 4πr^2^, divided by the number of source neutrons. This normalization gives unity in an unscattered, unattenuated environment and is useful for comparisons.

For the bare ^252^Cf source and the AmBe source, there is only a point neutron source in the no-walls geometry. For the D_2_O source, the 0.91 results from neutron attenuation and scattering in the D_2_O sphere. The full-geometry column includes scattering from the walls, etc. These numbers are greater than unity because they include both the direct and the scattered neutron fluence.

Agreement between measured and calculated results is good in all cases. The AmBe ratio is an average of ratios made at two distances, a ratio of 1.18 at 76 cm and ratio of 0.90 at 162 cm.

## 7. Summary

ROSPEC is a useful monitor for neutron fields. It gives a complete measurement of the neutron spectrum from thermal neutron energy up to 17 MeV. Since the measured results are not dependent upon fundamental physical parameters, ROSPEC is not suitable for the calibration of standard or reference neutron fields. However, it does verify the neutron spectrum for NIST calibration sources in various geometries, gives a measurement of scattering effects, and can be used to correlate neutron environments encountered in the field against standard or reference environments.

## Figures and Tables

**Table 1:** ROSPEC detector characteristics

Detector	Radius (cm)	Fill Gas	Pressure (kPa)	Energy Range
^3^He (bare)	2.54	^3^He/Kr	83	0.01 eV – 1 eV
^3^He (boron)	2.54	^3^He/Kr	216	1 eV – 0.05 MeV
SP2–1	2.54	H2	76	0.05 MeV – 0.25 MeV
SP2–4	2.54	H2	400	0.15 MeV – 0.75 MeV
SP2–10	2.54	H2	1000	0.4 MeV – 1.5 MeV
SP6	7.62	CH_4_/Ar	500	1.2 MeV – 5.0 MeV
SSS	2.8 cm radius	N/A	N/A	4 MeV – 16.6 MeV

**Table 2:** Calibration constants

Detector	Manufacturer Neutron Sensitivity	NIST Neutron Sensitivity
Bare ^3^He	0.178	0.586
Boron-covered ^3^He	0.00299	0.00583

**Table 3:** Measured and calculated neutron fluences

Field	Calculated No walls (n/S_n_)	Calculated Full Geometry (n/S_n_)	Measured (n/S_n_)	Ratio (Measured / Calculated) (Full Geometry)
Bare ^252^Cf (100 cm)	1.00	1.12	1.19	1.06
D_2_O Moderated ^252^Cf (100 cm)	0.91	1.05	1.05	1.00
AmBe (76/162 cm)	1.00	1.15 / 1.67	1.36 / 1.50	1.05

## Footnotes

1 Certain commercial equipment, instruments, or materials are identified in this paper to foster understanding. Such identification does not imply recommendation or endorsement by the National Institute of Standards and Technology, nor does it imply that the materials or equipment identified are necessarily the best available for the purpose.
